# A network meta-analysis of comparison of operative time and complications of laparoscopy, laparotomy, and laparoscopic-assisted vaginal hysterectomy for endometrial carcinoma

**DOI:** 10.1097/MD.0000000000010474

**Published:** 2018-04-27

**Authors:** Ya-Ru Wang, Hui-Fang Lu, Hui-Can Huo, Chang-Ping Qu, Gui-Xia Sun, Shi-Qing Shao

**Affiliations:** Department of Gynecology and Obstetrics, Huaihe Hospital of Henan University, Kaifeng, China.

**Keywords:** bowel injury, endometrial carcinoma, network meta-analysis, operative approach, operative time, randomized controlled trial, wound infection

## Abstract

**Background:**

The endometrial carcinoma (EC) is the most frequently occurring female genital cancer. The authors performed this network meta-analysis to compare operative time and the incidence of bowel injury and wound infection of 3 operative approaches (laparoscopy, laparotomy, and laparoscopic-assisted vaginal hysterectomy [LAVH]) in the treatment of EC.

**Methods:**

The Cochrane Library, PubMed, and Embase databases were searched. Randomized controlled trials (RCTs) for EC from the day of databases establishment to February 2017 were included. Direct and indirect evidences were combined to calculate the combined weighted mean difference (WMD) or odd ratio values and the surface under the cumulative ranking curve (SUCRA) value of 3 operative approaches in the treatment of EC.

**Results:**

A total of 9 qualified RCTs were included into the study. The results showed that laparotomy had a shorter-operative time than LAVH (WMD = −40.36, 95% confidence interval = −75.03 to −2.57). However, there was no significant difference in the incidence of bowel injury and wound infection among 3 operative approaches. Besides, the SUCRA values indicated that laparotomy had the shortest operative time but the incidence of bowel injury and wound infection was relatively higher.

**Conclusion:**

The results from this study indicate that laparotomy had highest incidence of bowel injury and wound infection but shortest operative time among 3 operative approaches in the treatment of EC.

## Abstract 2

Supplemental Digital Content is available in the text

## 1 Introduction

Endometrial carcinoma (EC), from the lining of the uterus, is the most frequent gynecologic malignancy of the female genital tract and the 4th most common neoplasia in women.^[1,2]^ As for EC, it is reasonable to expect a rise in the incidence due to life expectancy increase, and the significant rise is predominantly because of a large increased incidence in women aged above 60 years.^[3,4]^ EC occurs in postmenopausal women most frequently, and exposure to unopposed estrogen is the most important risk factor.^[5]^ As vaginal bleeding is commonly correlated with the disease, more than 75% of patients with EC are diagnosed at an early stage, contributing to overall favorable prognosis; therefore, the 5-year overall survival rate reaches to 80% to 85% and the survival rate reaches to 90% to 95%.^[6]^ The treatment for EC is primarily surgical in operable patients, and surgery is usually curative and is the mainstay of initial treatment for most patients with EC.^[7]^

Currently, the commonly used operative approaches for EC include laparoscopy, laparotomy, and laparoscopic-assisted vaginal hysterectomy (LAVH). Laparoscopy is used primarily as a diagnostic rather than a therapeutic procedure, and it has become the advisable method of access for certain gynecological interventions due to its infancy, which has been claimed to be associated with lower rates of adhesion development.^[8]^ Laparotomy is a commonly known high-risk surgical procedure, but with few data on postoperative care and few outcome data.^[9]^ Laparotomy is frequently performed for benign gynecologic conditions with the presence of a large distorted uterus and/or severe extrauterine diseases such as pelvic endometriosis or adhesion; operative injury to adjacent organs is a major concern of it.^[10]^ LAVH, firstly reported in 1989, is regarded as a feasible and safe technique,^[11]^ it mainly decreases pain, hospital stay, and surgical site infections, and led to a fewer postoperative adhesions and quicker return to normal activities.^[12]^ LAVH for treatment of endometrial cancer is related to lower perioperative morbidity in comparison to the conventional abdominal approach.^[13]^ Laparoscopy is a safer and more reliable surgical approach when compared with laparotomy in EC patients, with significantly decreased hospital stay and postoperative complications.^[14]^ Quality of life improvements from recovery, and adverse event profile, favor laparoscopy compared with laparotomy for treatment of EC in stage I.^[15]^

Nevertheless, there was little comprehensive and comparative analysis on the efficacy and complications caused by the 3 above-mentioned operative approaches in treatment of EC. In the present study, 3 different operative approaches (laparoscopy, laparotomy, and LAVH) for EC were searched out from relevant databases to compare their operative time and complications by performing this network meta-analysis.

## 2 Materials and methods

### 2.1 Ethics statement

Ethical approval and informed consent are not required, as the study will be a network meta-analysis and will not involve direct contact with patients or alterations to patient care.

### 2.2 Literature search

From the inception to December 2017, Cochrane Library and PubMed databases were performed by the computer-based retrieval combined with manual retrieval of related references of EC. With the combination of key words and free words, the search terms included EC, operative approach, laparoscopy, laparotomy, and LAVH. The PubMed database was taken as an example, and the detailed retrieval type could be seen in the attachment (search strategy—PubMed).

### 2.3 Inclusion and exclusion criteria

Inclusion criteria were as follows: study design should be randomized controlled trials (RCTs); operative approaches included laparoscopy, laparotomy, and LAVH; the age of EC patients should be from 27 to 89 years old; outcomes included operative time, incidences of bowel injury, and wound infection. Exclusion criteria were as follows: the studies lack data integrity; non-RCTs, non-English studies, nonhuman studies; and duplicate studies, conference reports, systematic reviews, and summaries.

### 2.4 Data extraction and quality assessment

With the standard data collection forms, data from included studies were extracted by investigators independently. Any disagreements were resolved through discussion. The risk of bias of included RCTs was assessed by 2 investigators according to the Cochrane Collaboration's tool.^[16]^ This tool included 6 items: random sequence generation, allocation concealment, blinding of participants and personnel, blinding of outcome assessment, incomplete outcome data, selective reporting, and anything else ideally prespecified. In the assessment, a judgment of “yes,” “no,” or “unclear,” each domain was assigned to designate, respectively, a low, high, or unclear risk of bias. If 1 or no domain was deemed “no” or “unclear,” the study was thought to have a low risk of bias. If 4 or more domains were deemed “no” or “unclear,” the study was regarded as a high risk of bias. If 2 or 3 domains were deemed “no” or “unclear,” the study belonged to a moderate risk of bias.^[17]^ Review Manager 5 (RevMan 5.2.3, Cochrane Collaboration, Oxford, UK) was used to carry out the quality assessment and investigation of publication bias.

### 2.5 Statistical analysis

First, traditional pair-wise meta-analyses were performed for studies to compare different treatment arms directly. The pooled estimates of weighted mean differences (WMDs) or odd ratios (ORs) and 95% confidence intervals (CIs) of EC were shown. I^2^ test and chi-squared test were used to test heterogeneity among the studies.^[18]^ Second, R 3.2.1 software was used to draw network meta diagram, in which each node represented a kind of intervention, the nodes size reflected sample size, and the thickness of lines between nodes meant number of included studies. Third, Bayesian network meta-analyses were performed to compare different interventions with each other. Each analysis was on the basis of noninformative priors for precision and effect sizes. Lack of autocorrelation and convergence were checked and confirmed by 4 chains and a 20,000-simulation burn-in phase; ultimately, direct probability statements were stemmed from an additional 50,000-simulation phase.^[19]^ The node-splitting method was adopted to evaluate the consistency between direct and indirect evidence. Based on the results of the selection of the consistency or inconsistency model, if the node-splitting showed *P* > .05, the consistency model was used to analyze.^[20]^ To assist in the interpretation of ORs or WMDs, researchers calculated the probability of each intervention to be the most effective or safest treatment method according to a Bayesian approach by using probability values summarized as the surface under the cumulative ranking curve (SUCRA), the larger the SUCRA value, the better the rank of the intervention.^[21,22]^ R (V.3.2.1) package gemtc (V.0.6) as well as the Markov Chain Monte Carlo engine Open BUGS (V.3.4.0) were used to do all computations.

## 3 Results

### 3.1 Baseline characteristics of included studies

**Figure 1:**
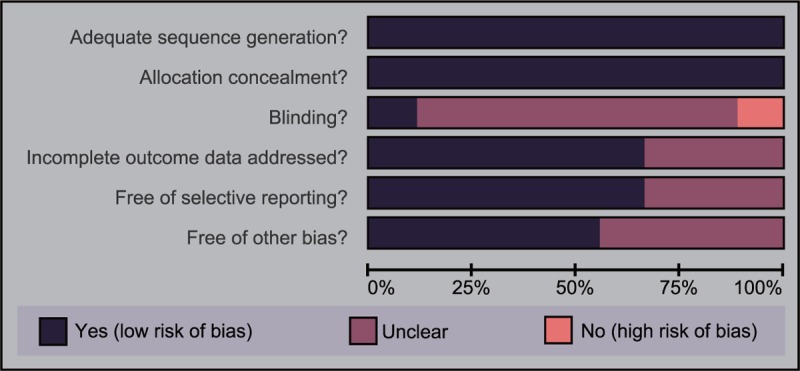
Cochrane system bias evaluation of included studies. Nine eligible randomized controlled trials are analyzed in this network meta-analysis.

**Figure 2:**
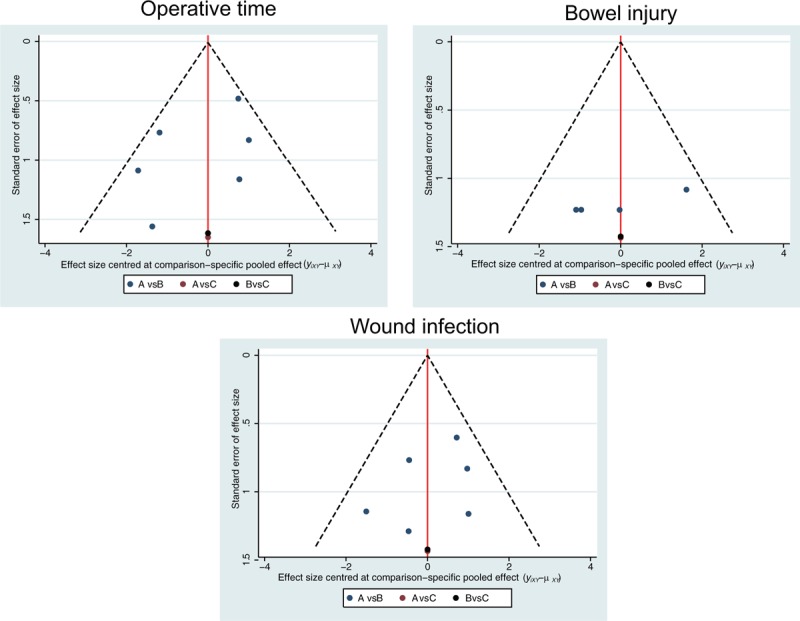
The funnel plot suggesting no existence of publication bias and studies included are of good quality. A = laparoscopy, B = laparotomy, C = laparoscopic-assisted vaginal hysterectomy.

A total of 711 relevant studies were initially identified, among that 7 studies were searched manually. We first excluded 5 duplicate studies, 72 letters and reviews, 87 nonhuman studies, and 64 non-English studies. After full-text review, of the rest 483 studies, 87 non-RCT studies, 152 unrelated to EC, 226 nonsurgical treatment, and 4 without data or data integrity were further ruled out. Finally, 9 RCTs were eligible to this meta-analysis^[13–15,23–28]^ (Supplementary Fig. 1). These studies included 2263 patients with EC. This network meta-analysis included 3 operative approaches: laparoscopy, laparotomy, and LAVH. The 9 RCTs were published from 2001 to 2013. There were 8 studies in Caucasians and 1 in Asians, and all the 9 RCTs were 2-arm trials. The baseline characteristics of included studies are displayed in Supplementary Table 1. The evaluation of risk of bias assessment of trials using the Cochrane Collaboration's tool of included studies is shown in Fig. 1. All included studies were in line with adequate sequence generation and allocation concealment. Only a few studies were confirmed that blind method was not adopted. Most of the literatures conform to complete outcome data addressed, free of selective reporting, and free of other bias. In addition, Fig. 2 reveals that the scattered points are located in the funnel and distributed evenly on both sides of the central line, which further shows that there is no obvious bias in the included studies. All in all, the quality of the research included is better, and the study was classified as having a low risk of bias.

### 3.2 Pair-wise meta-analysis for operative time and the incidence of bowel injury and wound infection of 3 operative approaches in the treatment of EC

Pair-wise meta-analysis results indicated that EC patients treated with laparoscopy had a longer operative time than those treated with laparotomy (WMD = 18.80, 95% CI = 5.10–32.49); and the operative time of EC patients treated with laparoscopy and laparotomy was relatively shorter than LAVH (WMD = −29.20, 95% CI = −49.03 to −9.37; WMD = −35.00, 95% CI = −57.09 to −12.91, respectively). However, there was no significant difference in the incidence of bowel injury and wound infection among 3 operative approaches (Table 1).

**Table 1:**
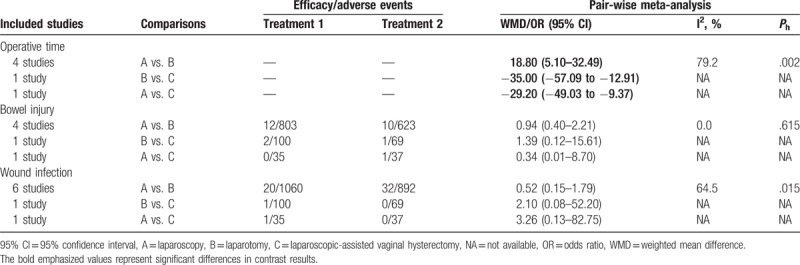
Estimated WMD/OR and 95% CI from pair-wise meta-analysis for efficacy/adverse events in endometrial carcinoma patients.

### 3.3 Evidence network of 3 operative approaches in the treatment of EC

**Figure 3:**
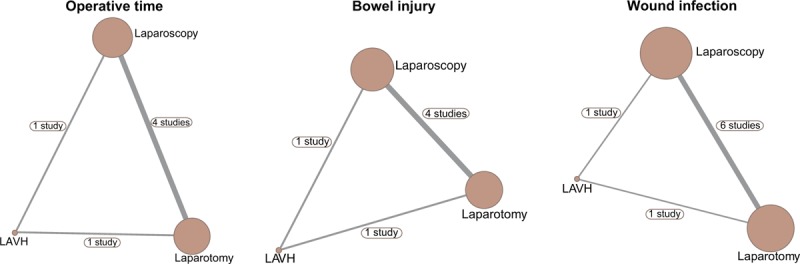
Network evidence graphs for operative time, the incidence of bowel injury, and wound infection. LAVH = laparoscopic-assisted vaginal hysterectomy.

In this network meta-analysis, the number of EC patients treated with laparoscopy was the largest. Meanwhile, direct-paired comparisons were relatively more about laparoscopy and laparotomy (Fig. 3).

### 3.4 Inconsistency tests of operative time, the incidence of bowel injury, and wound infection in 9 RCTs

Inconsistency tests showed that the results of the direct and indirect evidence of all outcomes were consistent, so the consistency model was adopted (both *P* > .05) (Table 2).

**Table 2:**
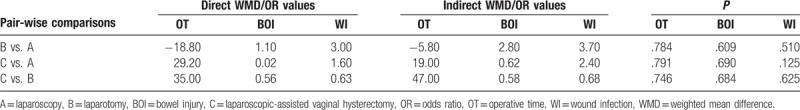
WMD/OR values and *P* values of direct and indirect pair-wise comparisons of 3 treatment modalities under 3 endpoint outcomes.

### 3.5 The main results of network meta-analysis for operative time and the incidence of bowel injury and wound infection of 3 operative approaches in the treatment of EC

**Figure 4:**
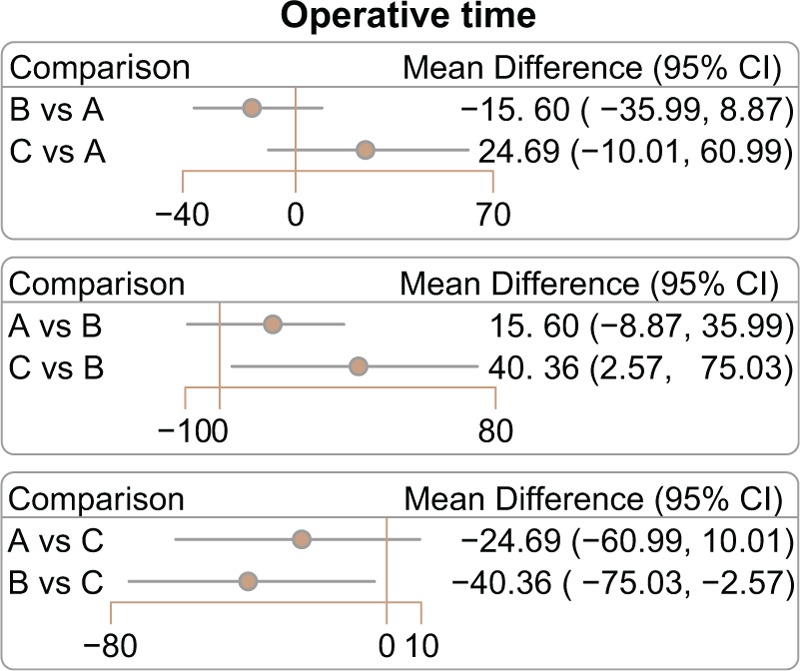
Relative relationship forest plots of the 3 surgical approaches as for operative time. A = laparoscopy, B = laparotomy, C = laparoscopic-assisted vaginal hysterectomy.

This network meta-analysis results indicated that EC patients treated with laparotomy had a shorter-operative time than those who treated with LAVH (WMD = −40.36, 95% CI = −75.03 to −2.57) (Fig. 4; Table 3). However, there was no significant difference in the incidence of bowel injury and wound infection among 3 operative approaches (Supplementary Fig. 2 and Table 3).

**Table 3:**
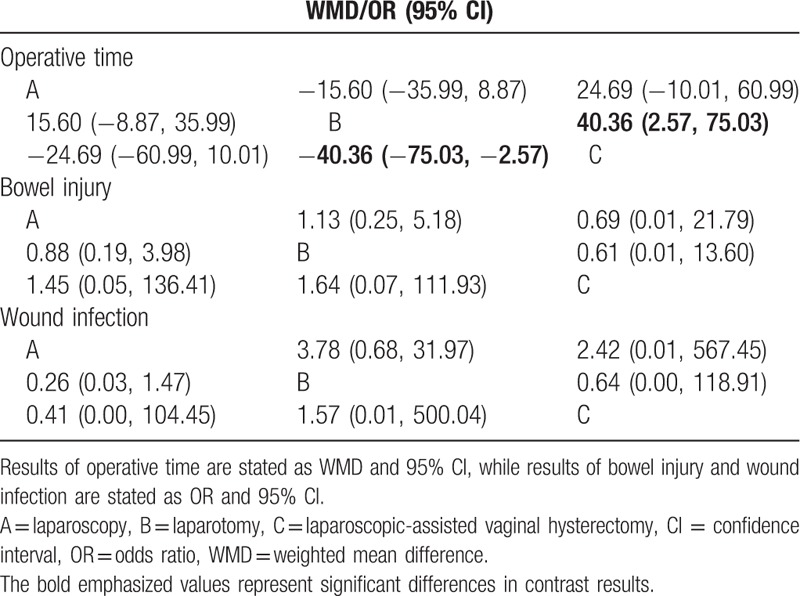
WMD or OR and 95% CI of 3 treatment modalities of operative time, bowel injury, and wound infection.

### 3.6 SUCRA values of operative time and the incidence of bowel injury and wound infection of 3 operative approaches in the treatment of EC

**Figure 5:**
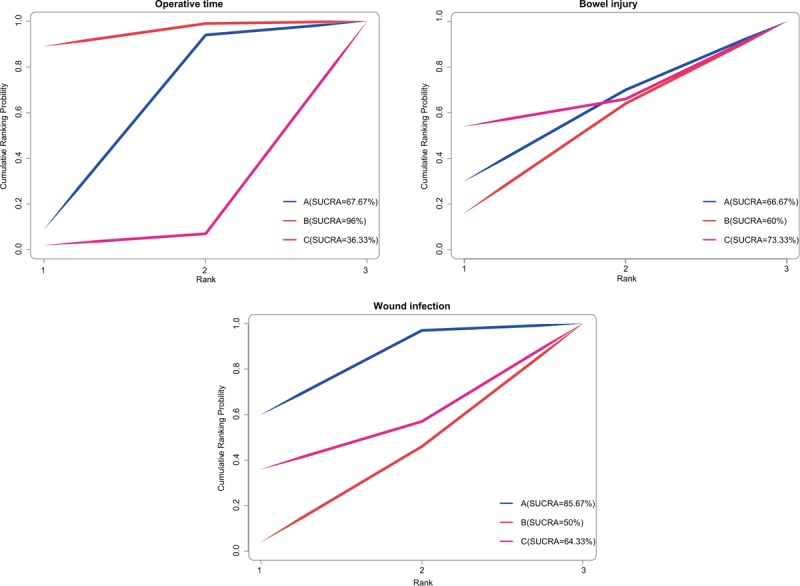
Diagrams of SUCRA values in terms of operative time, the incidence of bowel injury, and wound infection. SUCRA = surface under the cumulative ranking curves.

As shown in Fig. 5, the results indicated that the SUCRA value of operative time of laparotomy was the highest (96.0%), and LAVH was the lowest (36.33%). LAVH had the highest SUCRA value on the incidence of bowel injury (73.33%), and laparotomy had the lowest (60.0%). In addition, the SUCRA value of laparoscopy in terms of the incidence of wound infection was the highest (85.67%), and that of laparotomy was the lowest (50.0%).

## 4 Discussion

In the study, the RCTs concerning 3 operative approaches (laparoscopy, laparotomy, and LAVH) for EC were included to perform a pair-wise analysis and network meta-analysis. We drew a conclusion that laparotomy had the shortest operative time but the highest incidence of bowel injury and wound infection in the treatment of EC.

Our study has shown that laparotomy has the shortest operative time for patients with EC. Laparotomy is defined as an abdominal hysterectomy with a 10 to 15 cm vertical incision in the abdominal wall, through which the standard operation is carried out, and with a 3- to 5-day hospitalization, usually followed by a 6-week recovery time for patients.^[29]^ Laparotomy is traditionally used to treat various benign conditions and gynecological malignancies that are technically challenging, such as great uterine weight, mild prolapsed, endometriosis, and pelvic adhesions.^[30]^ As laparotomy is a traditional approach, the shorter-operative time may attribute to surgeons’ efficiency from years of practice.^[31]^ Ottosen et al proposed that the mean duration of surgery was significantly shorter for laparotomy compared with LAVH and vaginal hysterectomy.^[32]^ The most important factor in prevention of ureteric injuries lies in the common sites of ureteral lesions and adequate knowledge of pelvic anatomy.^[33]^ A study has shown that rate of ureteral injuries associated with laparoscopy was 35-fold higher than with laparotomy.^[34]^ Nevertheless, patients undergoing laparotomy inevitably suffer significant postoperative pain.^[35]^

Our findings indicated that laparoscopy and LAVH had the lower incidences of bowel injury and wound infection than laparotomy. Laparoscopy uses a lighted and thin tube putting through an incision in the belly to look at the female pelvic organs or the abdominal organs,^[36]^ has enjoyed wide acceptance in general surgery field, and numerous procedures have been developed for this minimally invasive approach.^[37]^ Laparoscopy offers a superior overview of the abdominal cavity with minimal trauma of the patient,^[38]^ with fewer wound complications, less postoperative pain, shorter hospital stays, and earlier ambulation.^[39]^ A previous study has demonstrated that laparoscopy is associated with significantly less use of pain medication, less blood loss, a faster recovery, and a shorter hospital stay than laparotomy.^[40]^ Another study showed that early and late postoperative complications rate of laparoscopy is lower than that of laparotomy.^[23]^ Nezhat et al has reported that the 2- and 5-year estimated recurrence-free survival rates for the laparoscopy are higher than those for laparotomy.^[25]^ In addition, laparoscopy has a shorter mean total operative time than LAVH, which means that patients with EC benefit more from laparoscopy than from LAVH in terms of shorter-operative time.^[27]^ Even though with greater total cost, those patients who undergoing LAVH have a greater rate of lymph node dissection, shorter hospital admissions, as well as similar postoperative morbidity and mortality.^[41]^ In addition, laparotomy showed better or equal results when compared with LAVH, thus it is more favorable compared to LAVH for removing the uterus with or without the adnexa in the lesions of the female genital system.^[42]^ With respect to the technical feasibility, LAVH represents a safe alternative for laparotomy when LAVH is contraindicated.^[43]^

This network meta-analysis comprehensively compares operative time and incidence of bowel injury and wound infection of different operative approaches in the treatment of EC. The integration of existing evidences provides a referential direction for the clinical choice for operative method in EC patients. However, there are still some limitations. First, the sample size of RCTs was generally small, which might add uncertainties to the evaluation. Second, only 3 indicators (operative time, bowel injury, and wound infection) were presented in this network analysis, due to which node-splitting analysis could not be performed, which may lead to a minor effect on the final results. Therefore, further studies with larger sample size and more indicators on operative approaches in the treatment of EC were conducted to verify our results.

In conclusion, this network meta-analysis suggests that laparotomy had higher incidence of complications than laparoscopy and LAVH in the treatment of EC, but laparotomy had the shortest-operative time among 3 operative approaches, which will provide a better basis for the treatment of EC.

## Acknowledgment

The authors would like to acknowledge the reviewers for their helpful comments on this paper.

## Author contributions

**Conceptualization:** Ya-Ru Wang, Hui-Fang Lu, Hui-Can Huo, Chang-Ping Qu, Gui-Xia Sun.

**Data curation:** Ya-Ru Wang, Gui-Xia Sun.

**Formal analysis:** Hui-Can Huo, Chang-Ping Qu.

**Investigation:** Hui-Can Huo.

**Methodology:** Hui-Fang Lu, Shi-Qing Shao.

**Validation:** Hui-Fang Lu, Shi-Qing Shao.

**Writing – original draft:** Ya-Ru Wang.

**Writing – review & editing:** Ya-Ru Wang, Hui-Can Huo, Chang-Ping Qu, Gui-Xia Sun, Shi-Qing Shao.

## Supplementary Material

Supplemental Digital Content
