# Supplementary material for: A network meta-analysis of comparison of operative time and complications of laparoscopy, laparotomy, and laparoscopic-assisted vaginal hysterectomy for endometrial carcinoma
Source: Medicine (Baltimore). 2018 Apr 27;97(17):e0474. doi: 10.1097/MD.0000000000010474 (PMC5944470; doi:10.1097/MD.0000000000010474)
Supplement: Supplemental Digital Content [file medi-97-e0474-s001.docx]

**Supplementary Table 1**

The baseline characteristics for included studies.

| **First author** | **Year** | **Country** | **Interventions** | | **Total** | **Number** | |  | | **Age (years)** | |  | |
| --- | --- | --- | --- | --- | --- | --- | --- | --- | --- | --- | --- | --- | --- |
|  |  |  | **T1** | **T2** |  | **T1** | **T2** | | **T1** | | **T2** | |  |
| Lu Q | 2013 | China | A | B | 272 | 151 | 121 | | 56.6 (27–82) | | 57.2 (29–79) | |  |
| Obermair A | 2012 | Australia | A | B | 753 | 404 | 349 | | 63 ±10 | | 63±10 | |  |
| Bijen CB | 2011 | Netherlands | A | B | 279 | 185 | 94 | | 62 (40–89) | | 63 (39–86) | |  |
| Janda M | 2010 | Australia | A | B | 332 | 190 | 142 | | 62.8±10.0 | | 62.7±9.7 | |  |
| Nezhat F | 2008 | USA | A | B | 194 | 67 | 127 | | 60±11 | | 63±11 | |  |
| Kalogiannidis I | 2007 | Belgium | B | C | 169 | 100 | 69 | | 66 (35-82) | | 63 (34-86) | |  |
| Ghezzi F | 2006 | Italy | A | C | 72 | 35 | 37 | | 63.5±8.8 | | 63±8.9 | |  |
| Tozzi R | 2005 | UK | A | B | 122 | 63 | 59 | | 67 (35–88) | | 66 (36–89) | |  |
| Malur S | 2001 | Germany | A | B | 70 | 37 | 33 | | ≈68.3 | | ≈67.7 | |  |

Note: T = treatment; A = laparoscopy; B = laparotomy; C = laparoscopic-assisted vaginal hysterectomy.

**Supplementary Figure 1.** Flow chart showing literature search and study selection. Nine randomized controlled trials met the inclusion criteria are included in this meta-analysis.

**Supplementary Figure 2.** Relative relationship forest plots of the three the three methods in terms of the incidence of bowel injury and wound infection.

Notes: A = laparoscopy; B = laparotomy; C = laparoscopic-assisted vaginal hysterectomy.
